# Self-assembling peptides-based nano-cargos for targeted chemotherapy and immunotherapy of tumors: recent developments, challenges, and future perspectives

**DOI:** 10.1080/10717544.2022.2058647

**Published:** 2022-04-09

**Authors:** Xue-Jun Wang, Jian Cheng, Le-Yi Zhang, Jun-Gang Zhang

**Affiliations:** a Department of General Surgery, Chun’an First People’s Hospital (Zhejiang Provincial People’s Hospital Chun’an Branch), Hangzhou, China; b General Surgery, Cancer Center, Department of Hepatobiliary and Pancreatic Surgery and Minimally Invasive Surgery, Zhejiang Provincial People’s Hospital (Affiliated People’s Hospital, Hangzhou Medical College), Hangzhou, China; c Key Laboratory of Tumor Molecular Diagnosis and Individualized Medicine of Zhejiang Province, Zhejiang Provincial People’s Hospital (Affiliated People’s Hospital of Hangzhou Medical College), Hangzhou, China

**Keywords:** Self-assembling peptides, morphology, nanocargos, chemotherapy, immunotherapy

## Abstract

Self-assembling peptides (SAPs) have enormous potential in medical and biological applications, particularly noninvasive tumor therapy. SAPs self-assembly is governed by multiple non-covalent interactions and results in the formation of a variety of morphological features. SAPs can be assembled in a variety of ways, including chemical conjugation and physical encapsulation, to incorporate multiple bioactive motifs. Peptide-based nanomaterials are used for chemotherapy, delivery vehicles, immunotherapy, and noninvasive tumor therapies (e.g. photodynamic therapy) by employing the self-assembling properties of peptides. The recent increase of SAPs is almost entirely due to their excellent biocompatibility, responsiveness toward tumor microenvironment, multivalency, and structural versatility. Synergistic therapy is a more effective and powerful approach to treat the tumor. Notably, SAPs can be used to subtly combine various treatments. Importantly, SAPs are capable of subtly making the combination of various treatments. This review describes mechanisms of peptides self-assemble into various structures and their biomedical applications with a focus on possible treatments.

## 1. Introduction

Peptides are amino acid chains made up of about 50 amino acids that are simple to produce and are even designed to mimic the self-assembly (SA) characteristics of proteins. Peptides have outstanding chemical diversity, high biocompatibility, and biological recognition capabilities. Furthermore, small peptides can translocate cell membranes but do not elicit an immunological response (Wang et al., 2019). Though, free peptides are usually unstable and undergo rapid degradation during the body's blood circulation, resulting in an off-target effect (Yang et al., 2018). Consequently, the elegant nanotechnology of the SA approach for modifying peptides and building stable and multifunctional nanomaterials has been developed in recent years specifically for tumor therapy (Yuan et al., 2019). SA is a necessary bottom-up method of construction in the toolkit of current nanotechnology. Today, SA is a growing field of research that incorporates concepts from supramolecular chemistry as well as contributions from chemistry, biology physics, and engineering. Notably, self-assembled materials have a wide range of applications in drug delivery, tissue engineering, electronics, and nanotechnology (Whitesides et al., 1991; Lehn, 2002).

A wide range of nanomaterials and therapeutic agents with enhanced architectures and functions have been designed and manufactured in the last several decades, thanks to the supramolecular (SA) peptides (Li et al., 2019b). To overcome some of the constraints inherent in molecular peptide immunotherapy, supramolecular peptide SA can produce nanostructures with increased stability, immune responses, and usefulness *in vivo*. Weak intermolecular interactions such as π–π stacking, hydrophobic interaction, hydrogen bonding, van der Waals and electrostatic interactions drive and control the creation of supramolecular peptide assemblies (Wang et al., 2016). When compared to the comparable molecular peptides, these assemblies show different immunogenicity or therapeutic properties. Self-assembling peptides (SAPs) are capable to form diverse nanostructures, including nanofibers, nanospheres, and micelles, which are commonly utilized in tissue engineering as well as in drug delivery (Tesauro et al., 2019).

**Figure 1.**
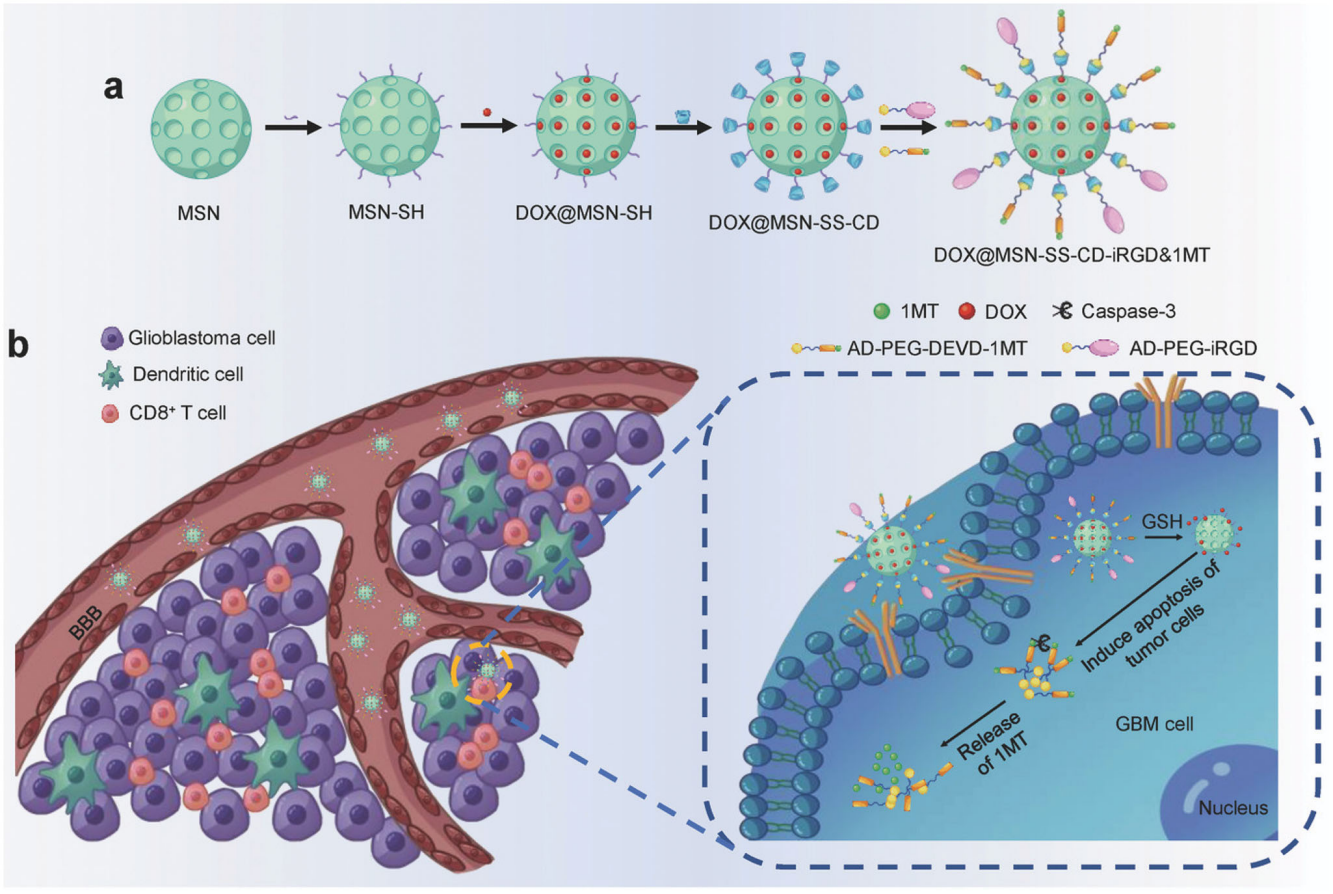
The mechanism of action of chemo-immunotherapeutic NPs. (a) DOX@MSN-SS-iRGD&1MT synthesis. (b). Schematic diagram of how DOX@MSN-SS-iRGD&1MT generated antitumor immunity against glioblastoma. The glioblastoma cell was targeted and the loaded drugs were released in a magnified image. Reproduced with permission from reference Lu et al. (2016).

Considering the unique features of supramolecular peptide assemblies, numerous researchers have been focusing on developing novel supramolecular techniques and optimizing their chemotherapeutic and immunotherapeutic efficacy (Zhang et al., 2019b). To form peptide-based self-assembled supramolecular nanomaterials with superior chemotherapeutic and immunotherapeutic capabilities, various requirements must be fulfilled (Yang et al., 2019): including (1) through SA, selection of easily available immunogenic peptides with enhanced immune performance and recognition epitopes. (2) Identifying peptides with a well-defined metabolic mechanism and chemical composition, as well as the ability to assemble them, to generate extremely biosafe nanodrugs with easy administration. (3) Developing nanostructures that are well-defined and stable, capable of loading therapeutic drugs and reducing drug clearance in the body. (4) Addition of responsive substances enables the TME to be changed and the immune response to be regulated flexibly. (5) Having the ability to specifically target tumor cells while minimizing toxicity to normal cells. Additionally, supramolecular assemblies of the peptide can be covalently or noncovalently integrated into functional photosensitive or chemotherapeutic drugs, allowing for the development of multifunctional nano DDS for combination immunotherapy (Chang et al., 2019). A deep understanding of supramolecular peptide assemblies' physicochemical characteristics and the mechanism by which they exert immunological properties are required for the design and implementation of supramolecular immunotherapeutic materials and agents (Figure 1).

Several studies have been published regarding SAPs and their applications in the biomedical field. This article summarizes the recent progress in the use of SAPs as immunotherapeutic and chemotherapeutic nanosystems and the mechanism by which supramolecular assemblies affect these nanomaterial's performance in terms of immune regulation and delivery carriers. In addition, the mechanisms and driving forces are discussed through which different peptides-based supramolecular assemblies including peptide-based nanoparticles, nanospheres, nanofibers, vesicles, and hydrogels are formed. Moreover, supramolecular assemblies of peptides are also discussed in detail for combination chemotherapy and immunotherapy.

## 2. Driving forces for self-assembly of peptides

Without the need for external intervention, highly organized nanostructures for DDSs are formed by molecules self-assembling (Lu et al., 2016). Secondary interactions including hydrogen bonding, hydrophobic and van der Waals interactions, coordination forces, steric and depletion forces, solvation, pi to pi interactions, and hydration forces all play a role in peptide SA (Fan et al., 2017). Hydrophobic interactions are responsible for pi to pi stacking in SA of peptides, which are made up of various hydrophobic amino acids. Polar amino acids are made up of charged functionalities that form hydrogen bonds and interact electrostatically. Furthermore, the peptide backbones provide hydrogen bonding, ensuring that the SA of peptides is more stable (Hiscock et al., 2016). All of these interactions have a significant impact on the formation of the nanostructures during peptide SA (Zhao et al., 2018). External stimuli such as temperature, pH, and solvent polarity have an impact on these noncovalent interactions. As a result, such stimuli can also cause peptides and nanostructures to self-assemble. Amino acids like arginine, histidine, lysine, and glutamine have been shown to respond to changes in pH. The pH values of the exposed environment have a significant impact on the SA of these peptides. Peptide nanostructures can only be stable if all noncovalent interactions are considered and applied in drug delivery applications (Sun et al., 2015).

### 2.1. Hydrophobic interactions

Among the numerous noncovalent interactions that contribute to the SA of peptides, hydrophobic interactions are considered the most important. The majority of SA building blocks are amphiphilic peptides that contain both polar and nonpolar parts and self-assemble readily via thermodynamic microphase separation processes. The nonpolar portions of the fundamental unit collapse and aggregate in an aqueous system to provide a barrier against water for the hydrophobic section. At the same time, the polar components make more interaction with the aqueous part (Raza et al., 2019). Hydrophobic interactions have permitted the incorporation of a variety of amphiphilic drugs into such self-assembled systems. One example included the conjugation of TAU protein with camptothecin (CPT), a hydrophobic anti-tumor drug. Through hydrophobic interactions and hydrogen bonding, the peptide may self-assemble into fibril structures (Wang & Gong, 2017).

### 2.2. Electrostatic interactions

Electrostatic interactions also play a role in the SA of the peptide. There may be both attractive or repulsive interactions depending on the charged moieties of amino acids exerting a strong influence on the SA process. Clusters are formed via the interaction of negatively charged peptides with the peptides carrying a positive charge. Additionally, electrostatic interaction also facilitates the incorporation of the drug into self-assembling charged peptides. Electrostatic forces form highly organized and stable nanostructures that can be used in a variety of DDSs (Hu et al., 2016). Electrostatic interactions have been used to form nanosystems with multifunctional characteristics using peptides such as KALA and cRGD-BSA cell-penetrating peptides. Due to the pH sensitivity of these nanostructures, they can be used in pH-responsive DDSs (Chen et al., 2015).

### 2.3. Hydrogen bonding

In nature, hydrogen bonding occurs in peptides' α-helices, β-sheets, and coiled-coil structures. It is also used to self-assemble various nanostructures to design various sequences of peptides (Lu et al., 2016). Additionally, hydrogen bonding plays a significant role in the peptides SA into network structures. The carbonyl and amide groups in the peptide backbone contribute to the hydrogen bonding, which is in turn associated with the peptide structure's stability. Peptides are typically composed of both hydrophilic and hydrophobic segments formed by repeating units of amino acid in a chain structure. In an aqueous environment, hydrophilic segments are found in water, whereas hydrophobic segments are hidden within the self-assembled network. In contrast to β-sheets, α-helices are produced through hydrogen bonding between the amide groups peptide backbone. Hence, side chains form on the surface of each α-helix, enhancing stability in an aqueous medium (Ulijn & Smith, 2008).

### 2.4. Pi to pi stacking

Pi to pi stacking is also critical for preserving the nanostructures formed via SA of numerous aromatic peptides. The pi to pi stacking of peptide structures is responsible for their directional growth (Fan et al., 2017). Due to the aromatic groups present in these structures, they have a low solubility in water (Wang et al., 2016). However, in organic solvents such as trifluoroacetic acid (TFA) and toluene, pi to pi stacking is more prevalent (Zhu et al., 2010). The nanostructures formed via SA of dipeptides such as diphenyl alanine (FF) are stabilized by interactions between the aromatic moieties such as pi to pi stacking and hydrogen bonding. Numerous DDSs have been proposed using self-assembled FF dipeptide nanostructures.

#### 2.4.1. Formation mechanisms of peptide assemblies

Peptides adopt a specific configuration when dissolved in a solvent and that configuration ultimately determines which self-assembled structure will be formed. In nature, self-assemblies are spontaneously formed during protein folding, the formation of DNA double-helix, and cell membranes formation (Habibi et al., 2016). Various nanostructures ranging from zero-dimensional (0D) nanospheres and nanoparticles to three-dimensional (3D) hydrogels and vesicles have been reported for peptides self-assemblies. The formation of advanced assemblies of peptides is governed mainly through different external and internal molecular interactions including H-bonding, electrostatic-interactions, hydrophobic-interactions, pi–pi interactions, aromatic stacking, and others (Wang et al., 2021b). These peptide-based assemblies have been extensively used in various biomedical application including cancer therapy and diagnosis (Wang et al., 2020). Knowledge regarding the mechanism of peptides’ self-assemblies and stability of constructed structures is vital for the design of functional materials (Wang et al., 2021b). The α-helices, β-hairpins, and β-sheets are the main secondary structures of peptides that prefer the formation of self-assembled structures. Linear peptides containing α-helix structures lose their helical conformation in solution due to their inherent thermodynamic instability (Liu et al., 2008). Thus, it is important to stabilize α-helix for triggering SA in peptides (Hu et al., 2018, 2019, 2020). Various approaches for the stabilization of α-helix include metal coordination (Ruan et al., 1990), side-chain cross-coupling, hydrogen-bond surrogates (Liu et al., 2008), and salt bridge formation (Marqusee & Baldwin, 1987).

#### 2.4.2. Zero-dimensional peptide nanoparticles and nanospheres

Peptide nanoparticles (PNPs) and nanospheres are examples of 0D peptide nanostructures that have a size range from 1 to 100 nm and are extensively used for various biomedical applications. Target-specific ligands can produce stimuli-responsive nanoparticles and could be ideally designed for the release of payload in the desired area of the body, i.e. tumor sites (Wang et al., 2021b). The driving forces for the SA of PNPs are the interactions between α-helices hydrophobic amino acids and ionic interactions between parallel dimers and trimers which stabilize coiled-coil interfaces (Doll et al., 2015). Emulsion polymerization in an aqueous medium is a handy method for the synthesis of 0D PNPs with the use of initiators, monomers, and surfactants. Polypeptide nanoparticles preparation with monomer emulsion polymerization of amino acids has been reported by Jacobs et al. (2019). The monomer which was UV light-sensitive was selected for the construction of PNPs. Subsequently, a block of glycosylation peptide was used for achieving aggregation of particles after cross-linking.

Another example of 0D peptide assemblies is peptide nanospheres that have attracted wider scientific interests due to their unique physicochemical stability and are extensively studied for genes and drugs delivery (Mumcuoglu et al., 2015). The presence of certain amino-acid residues, i.e. arginine and tryptophan in the peptide sequence can act as stabilizing agents and help in the construction of stable self-assemblies (Mandal et al., 2013). Hydrophobic interactions and H-bonding play a key role in the formation of 0D peptides nanospheres. Panigrahi et al. recently reported peptide nanospheres from L,L-cyclic peptides using hydrophobic (Trp), cysteine (Cys), and positively charged (Arg) residues which turned out to be an excellent vehicle for VEGF siRNA and VEGF antisense oligonucleotides’ intracellular delivery (Panigrahi et al., 2022). The *in silico* analysis of peptides SA showed that hydrophobic interactions and H-bondings were mainly involved. The π–π stacking in peptide molecules is also important for the formation of nanospheres. A diphenylalanine peptide self-assembles into 0D nanospheres due to its molecular structure which is more rigid with less degree of freedom, steric hindrance, and restricted rotation around C–C bonds. Fullerene-like nanospheres formation has been reported for cysteine diphenylalanine tripeptide (Reches & Gazit, 2004). In another study, a single-component based drug delivery system using the Fmoc-L-Trp-L-Phe-OCH3 framework has been reported (Singh et al., 2020). Pi–pi stacking and hydrophobic interactions between dipeptides were found to be responsible for the SA process.

#### 2.4.3. One-dimensional (1D) peptide nanofibers and nanotubes

Peptide amphiphiles with hydrophilic or charged amino acids linked to hydrophobic lipid or alkyl chains and β-sheet sequence favor the formation of 1D nanotubes and nanofibers (Rubert Pérez et al., 2015). Upon exposure of the alkyl chains to the aqueous environment, they tend to shield themselves from water molecules and avoid unfavorable interaction thus, turn into a self-assembled 3D structure including nanofibers (Matson et al., 2011), and nanotubes (Habibi et al., 2016). Several studies have been reported on β-sheets based SA of peptides suggesting its role in the formation of 1D nanostructures. Peptides containing hydrophobic and hydrophilic surfaces, e.g. Lego peptides (Zhang et al., 1993, 1994, 1995) can form β-sheet structures due to H-bondings in an aqueous medium. Hydrophobic collapse and β-sheet formation induce the formation of 1D nanostructures including ribbon or cylindrical-shaped nanofibers (Hendricks et al., 2017). β-sheet mediated SA of peptides can stabilize α-helix, thus promoting the SA of peptides to nanostructures in an aqueous environment (Lim et al., 2009b). Li et al. constructed coiled nanofibers from five amino acids containing peptides scaffold with a thioether containing side chain (Hu et al., 2016; Jiang et al., 2021). RADA16-I peptide made of aspartic acid, alanine, and arginine form β-sheet structure and self-assembles into nanofibers. The alanine residues assemble together in an aqueous environment to decrease the energy of the system and gain stability, while the arginine and aspartic acid residues attract each other via electrostatic interactions and arrange themselves in the outer layer toward the aqueous environment. The alanine fragments slide laterally to decrease their association with the aqueous environment, and ultimately form the hydrophobic surface and completely fit to form a regular β-sheet structure (Yokoi et al., 2005).

β-hairpin structure containing peptides has been reported to favor 1D nanofibers. β-hairpin is a derivation of β-turn, that requires a peptide chain portion with a bendable amino acid sequence. MAX1-7 peptides designed by Schneider et al. can produce β-hairpin morphology (Schneider et al., 2002). Typically, these peptides are made of alternating hydrophilic (lysine-residues) and hydrophobic (valine-amino) sequences. These peptides form β-hairpin structures when pH or the ionic strength of the solution is increased, the lysine residues form the inner surface while valine residues make the outer layers shield electrostatic repulsion. Further utilization of hydrophobic interactions transforms these peptides into self-assembled nanofibers (Schneider et al., 2002; Lamm et al., 2005; Veerman et al., 2006).

In nanotubular structures formation, molecular interactions play a major role. The driving force for maximum stability and minimal surface area spontaneously take place and turn the peptides into cylindrical shapes once stacking took place. The SA of amino acid molecules can also be mediated by the synergistic forces of water molecules. To achieve structural stability, water molecules interact with the walls of constructed nanotubes and other solvents (e.g. ethanol) with the help of H-bondings. The interactions between charge ends and amino acid side chains also contribute to originating the process of SA. In short, the stacking interactions followed by SA to attain minimum energy of the system leads to the formation of 1D nanostructures (Wang et al., 2021b).

#### 2.4.4. Two-dimensional (2D) peptide nanosheets and nanobelts

The molecular SA and programming of peptides with defined sequences provide important avenues for the construction of 2D peptides’ based assemblies including nanosheets and nanobelts. Peptides can be arranged in well-defined supramolecular assemblies due to their high-density chemical functions. The information at the molecular level could serve to promote highly specific intermolecular and intramolecular interactions in a specified environment and construct structure-defined and thermodynamically stable 2D SA materials (Wang et al., 2021b). The disulfide-bond formation, π–π stackings, and metal ions coordination have been employed for the construction of collagen-mimic peptides (CMPs) based supramolecular assemblies, including nanospheres, discs, and fibers. Studies have shown that induction of structural modifications in CMPs could produce complex patterns of natural collagen assemblies. However, because the initial peptide sequence cannot consistently predict the order of assembly, the final structure frequently did not reflect the structural hierarchy of natural collagen isotypes. Recent research has achieved breakthroughs in several areas for the construction of peptides-based 2D assemblies. Two collagen-like peptide sequences, i.e. NSI and NSII were designed by Jiang et al. which self-assembled into nanosheets with defined structures (Jiang et al., 2014). The layered stacking of 2D collagen triple helices was the main underlying mechanism for designed nanosheets formation. Nanobelts and other supramolecular assemblies can be created using the SA nature and application of other stimuli to certain peptides (Cui et al., 2009). For example, hydroxyapatite (HAp) nanostrips were prepared via mild microwave heating of the precursor solution and adding cationic surfactant CTAB as a soft template for growth process and nucleation. The constructed nanostrips showed nearly 10 nm length and 55 nm width (Arami et al., 2009). The peptides’ concentration in solution also affects the fabrication of various peptides-based assemblies. For instance, narrow and twisted nanoribbons formation has been reported at low peptides concentration while nanoribbons have been observed at high peptide concentration. Similarly, the structure of nanobelts is also influenced by the pH values of the solution. When the pH value of the solution is increased, grooved nanoribbons were formed from flat nanoribbons and the opposite was observed at low pH (Cui et al., 2009).

#### 2.4.5. Three-dimensional peptide vesicles and hydrogels

Vesicles are flexible 3D closed structures with spherical architectures. Hollow spherical vesicles are usually single-layered or double-layered membranes made of synthetic or natural amphiphilic building blocks and have shown excellent applications in genes/drugs delivery and other biomedical fields (Haridas, 2021). Substantial development, especially in stimuli-responsive applications of vesicles has been made in recent years. However, peptides usually assemble into 1D and 2D structures, and reports regarding peptide-based vesicles are occasional (Jiang et al., 2014). Many factors restrict peptides aggregation into vesicles including an abundance of hydrogen bonds imposing directionality, planarity of peptide bonds restricting the flexibility of polypeptide chains, and chirality of amino acids. Macrocyclic peptide blocks have been prepared by Jeong and Lim which self-assembled to form peptides-based vesicles having abilities of molecular recognition (Jeong & Lim, 2014). The peptide building blocks self-assembled into vesicles at an extraordinary hydrophilic to total mass ratios contrary to conventional amphiphile molecules. The SA of peptides into vesicles requires flexibility in a lipophilic portion of peptides. To achieve this and increase π–π interactions, Jeong and Lim inserted the glycine in the middle portion of the lipophilic segment instead of the N-terminal region. This replication of only a single (glycine) residue considerably altered the nano-structure uniformity and morphology. These results suggested that implanting the bendable glycine enhanced the overall flexibility of the lipophilic segment, which along with reduced mobility of the peptide, strengthened internal lipophilic packing of the assembly.

Hydrogels are a class of materials mainly composed of low-molecular weight cross-linked molecules or polymers having the ability to accommodate a high quantity of water or aqueous solutions keeping their unique 3D structure (Kopeček & Yang, 2012). It has received wider research attention in recent years due to its applications in drug delivery, tissue engineering, and water treatment. The adjustable characteristics and higher water contents of hydrogels render them appropriate synthetic mimics for soft tissues microenvironment along with excellent media for drug delivery and local storage (Narayanaswamy & Torchilin, 2019). Peptides possess the advantages of high versatility, biocompatibility, and secondary adjustable structure. In particular, the secondary structure, i.e. α-helix β-sheet conformation could be exploited as a driving force for the formation of uniform fiber structure and subsequent assembly into covalently crosslinked 3D networks that can retain aqueous media to construct hydrogels. Peptides have been thoroughly used for the development of hydrogels as versatile building blocks (Kopeček & Yang, 2012; Pashuck, 2018). It has been reported that β-amino acids derivatives self organize to form hydrogels and show extended bioavailability in comparison with α-amino acid derivatives (Lee et al., 2019). The driving force for peptide self-assembled vesicles could be the hydrophobic segments that initially form ordered bilayer structures and ultimately spherical water-filled vesicles (Gudlur et al., 2012).

## 3. Self-assembly of peptides with a designed primary and secondary structure

According to the Structural Classification of Proteins (SCOP) database, natural proteins now have 1393 distinct folds (Lo Conte et al., 2000). When it comes to the construction of artificial peptides, β-sheets and helices are among the most commonly used secondary structural elements. Another secondary structure, random coil, indicates that the peptide lacks any hydrogen-bonding driven intramolecular structure. In nature, elastin-like peptides (ELPs) are a significant class of these peptides that are made from tropoelastin, the natural elastin precursor. Five amino acids are found in ELPs (i.e. pentad) repeating unit of Val-Pro-Gly-X-Gly sequence, where X is a guest residue (other than Pro) affecting the physical features of the assemblies of peptide, including lower critical solution temperature (LCST) and flexibility. Thermally responsive hydrogels containing elastin-mimetic or ELPs have been synthesized for tissue engineering and stimuli-responsive gene delivery (Meco & Lampe, 2019; Shmidov et al., 2019).

### 3.1. Primary structure

Peptides, in the absence of intramolecular structure, act as sequence-controlled, biomimetic random coil polymers, which facilitates the process of SA. SAPs with no secondary structure are the subject of several emerging research fields, including intrinsically disordered peptides (IDPs) and coacervates that was based on peptide (Blocher & Perry, 2017; Kaminker et al., 2017; Uversky, 2019). Key features like liquid–liquid phase separation behavior, processing, and coacervate droplet formation are all influenced by the primary structure of these peptides (Sing & Perry, 2020). These investigations have sparked widespread interest in the construction of sequence-controlled polymers, such as polymer ionic liquids and polyampholytes, that exhibit tunable phase behavior, structure, and desired properties (Delaney & Fredrickson, 2017; Ejeromedoghene et al., 2021).

### 3.2. Secondary structure

Peptides, capable of forming β-sheet, have been extensively employed to produce filamentous or fibrous stochastic assemblies such as helical tapes and β-strands (Zhang, 2003). The peptide backbone is stretched in a β-strand, and the hydrogen bonding groups face the peptide chain in an orthogonal direction. A β-sheet is formed as a result of the lateral joining of β-strands by hydrogen bonding (Vincent et al., 2013). The hydrogen bonding between the amino acids in different strands of peptide forms the sheet-like structure. The interpeptide and interchain bonds significantly increase the structure's stiffness (Boyle & Woolfson, 2011). Peptides that form β-sheets and form supramolecular SA are typically roughly 16–20 amino acids long, with alternating patterns of hydrophobic and polar amino acids. Strand alignment in β-sheets can be parallel or antiparallel, resulting in differing hydrogen binding patterns for these two types. Computational analyses revealed that antiparallel β-sheets are more energetically preferred than parallel forms due to the highly aligned hydrogen bonds (Perczel et al., 2005). The high hydrophobic side chain concentration (>50%) of this secondary structure type leads to their potential to undergo irregular aggregation or hierarchical assembly on β-sheet formation, indicating the examples of secondary structure-based deterministic assembly (Matsuurua, 2014; Levin et al., 2020). In addition to the previously mentioned peptide conjugates and peptide amphiphiles with other SA molecules, which adopt various nanostructures such as cylindrical and spherical micelles, vesicles, helical tapes, and fibrils, other important classes of empirically designed β-sheet-former peptides can be found in this category. These molecules possess important applications in various fields including inorganic timeframe, drug delivery, and tissue engineering (Arango-Restrepo et al., 2019; Dasgupta & Das, 2019). These peptide-like surfactants have been studied extensively for their self assembling capability, along with natural and synthesized anti-microbial peptides (AMPs) that are known to form β-sheets (Lombardi et al., 2019).

α-Helices are another type of secondary structure found in proteins, in which the amino acids form hydrogen bonds between the carbonyl oxygen and the hydrogen in every third amide group, thereby stabilizing the peptide backbone. The amino acid side chains extend outward from the α-helix's outer surface (Dieckmann et al., 2003). There are not many examples of helical peptide amphiphiles since they are difficult to keep stable and are usually on the edge of being stable. It is very important to balance the length of the alkyl polymer, spacer, and the length of the peptide headgroup to keep helical peptide amphiphiles stable. Liu et al., for example, demonstrated that peptide amphiphile containing three or four heptad repeat sequences of palmitic acid tail conjugated IEEYTKK are assumed to have the helical conformation and are capable to self-assemble into vesicles or spheres (Meng et al., 2014). Tirrell et al. conjugated a peptide derived from residue 1419 of the tumor suppressor protein P53 and the W3K sequence with a C16 alkyl chain to form nanoribbons or spherical micelles, respectively, to demonstrate another unique instance of helical peptide amphiphiles (Missirlis et al., 2011; Shimada et al., 2012). When P53 peptides were used, it was discovered that the linker between the alkyl chain and the peptide affected the extent of helical packing, with the addition of a tetra-alanine motif transforming the α-helix rich nanoribbon assemblies into β-sheet rich core–shell worm-like micelles (Missirlis et al., 2010). In the case of the W3K sequence, aging and processing resulted in a structural shift toward β-sheet assemblies (Shimada et al., 2011).

Another significant advance was the use of a covalently connected dimer of pentapeptides, formed by strategically placed cysteine groups, to stabilize the oligopeptides' helical structure and promote SA into 2D sheets at the air–water interface (Lee et al., 2016). There have also been reports of helical peptide-based surfactants (Xue et al., 2014; Braide-Moncoeur et al., 2016). Finally, SA of single peptide-like helical molecules has been demonstrated using multiple artificial peptide-like foldamers with a high proclivity for forming stable α-helices (Rinaldi, 2020).

## 4. Advantages of self-assembling peptides

### 4.1. High biocompatibility

For nanomaterials to be used in clinical therapy, they must be biocompatible. As peptides are derived from parts of natural proteins, they contain a variety of essential amino acids for human health. Naturally, peptides have excellent biocompatibility, hence they are preferred for use in the biosynthesis of nanoparticles (Wang et al., 2016). With SAPs, there are no intractable issues of toxicity or degradation resistance that other inorganic nanomaterials might have to contend with. For instance, the rapid degradation of zeolitic imidazolate framework-8 nanoparticles results in a high level of toxicity, while silica nanorattles are difficult to metabolically degrade (Su et al., 2019). Multifunctional nanomaterials based on SAPs exhibit greater biocompatibility than free hydrophobic drugs, thereby facilitating tumor therapy more effectively (Peng et al., 2019). SAPs nanomaterials exhibit a high degree of biocompatibility, which is critical in biomedicine.

### 4.2. Tumor microenvironment response

The tumor microenvironment (TME) presents different obstacles, thus preventing the nanomaterials transportation into the tumor. This in turn limits their applications in the treatment of tumors (Wong et al., 2020). Increased retention and permeability alone are insufficient to overcome this obstacle. Furthermore, the characteristics of the TME are frequently variable, depending on the location, type, and stage of progression of the tumor. Each microvasculature has a unique distribution of pore sizes; for example, pancreatic tumors may be 50–60 nm in diameter, while brain tumors may be 7 nm in diameter; thus, the design and development of nanomaterials with the appropriate size distribution is critical for effective tumor therapy (Rinaldi, 2020). According to some reports, the size of the nanomaterials determines their penetration depth into tumor tissue, with smaller nanoparticles penetrating deeper (Chauhan et al., 2012). However, extremely small particles (<11 nm) are rapidly cleared, which is detrimental to tumor therapy (Choi et al., 2010). It is worth noting that by varying the co-solvents, ionic strength, temperature, and pH of peptides, they can self-assemble into nanomaterials of varying sizes, which is advantageous for permeation into TME (Rinaldi, 2020). Additionally, non-spherical nanomaterials such as disc-shaped and rod-like nanoparticles penetrate and accumulate more rapidly in tumor sites than spheres of various sizes, and are suitable for tumors with smaller vessel-pore sizes due to the particles' shortest dimension (Chauhan et al., 2011). Thus, peptides are an excellent choice because they can self-assemble into nanomaterials with the desired morphology for specific tumors via noncovalent interactions.

### 4.3. Multivalency

Multivalency is a critical property of self-assembling nanostructures, as it enables the formation of multivalent interactions, which improve the binding affinity of weakly specific interactions (Lim et al., 2009a). Self-assembling peptides have the important property of multivalency because peptides produce self-assembling nanoparticles by a bottom-up self-assembling process. Immune system activation can be caused by multivalent antigens recognized by B cells, which are important in bioactive functionalization (Puffer et al., 2007). Furthermore, in biological systems, multivalency plays a significant role in enhancing avidity and specificity, whereas monovalency does neither as well. The associativity of receptors can be improved by reorganizing some of the receptors on the cell surface into multivalency. Thus, multivalency SAPs can be used to activate immunogenicity and promote immunotherapy for tumors (Rudra et al., 2012). Vaccines and vaccine adjuvants against tumor cells are currently being developed using SAPs with multivalency (Collier, 2008). SAPs have a unique advantage in immunotherapy and other fields because of their multivalency (Liu & Kiick, 2008).

### 4.4. Diverse structure

Peptides can self-assemble into a variety of nanostructures in an aqueous solution under a variety of environmental conditions (Chen & Rosi, 2010). For example, when peptides are dissolved in a low-pH and high-osmotic pressure solution, nanofibers form rapidly. To rationally design objective structures, it is therefore beneficial to understand the structures of SAPs and the mechanism of SA (Zhao et al., 2019). Certain SAP structures have been reported to be stable, which makes them suitable for biological applications. For example, the coiled-coil structure is more stable than other ɑ-helices (Rad-Malekshahi et al., 2016). Numerous SAPs structures have a variety of bioapplications. According to studies, the structure of nanomaterials can affect the recognition and uptake of cells, as well as the immune response (Branco et al., 2011). When compared to hydrogels and β-sheet fibrils formed by peptides, nanofibers potentially facilitate the attachment, differentiation, and growth of different types of mammalian primary cells. Similarly, the fibrillated peptides can effectively enhance the responses of the antibodies, thus resulting in the production of specific antibodies without supplying any immune adjuvants (Davis et al., 2005; Yang et al., 2020b).

## 5. Self-assembling peptides-based cargos

Due to their high biocompatibility and chemical versatility, SAPs can be used as cell and drug delivery vehicles (Moyer et al., 2014). In recent years, the field of medicine has seen a surge in the use of delivery cells, also known as cell transplantation. Cell transplantation is being used to treat an increasing number of diseases (Davis et al., 2005). Current methods, on the other hand, have several flaws, such as a low transplanted cell survival rate and a lack of a proper delivery system with oxygen and nutrients. Due to their controlled architecture and dimensions, SA nanomaterials, in particular, provide a living space for bioactive signals and cells (Webber et al., 2010). Thus, SAPs are thought to be suitable carriers for transporting cells, and they have even been used to build tissue-specific models *in vitro* and reconstruct TMEs in 3D cell cultures for tumor therapy (Yang et al., 2020b). Lisa et al. reported that peptide-based hydrogelation can make cells distribute homogeneously in hydrogels while maintaining cell viability for cell transplantation and targeting biological sites (Haines-Butterick et al., 2007). SAPs with a skeletal structure capable of supporting cells' living space have a greater potential for cell delivery in the field of medicine.

**Figure 2.**
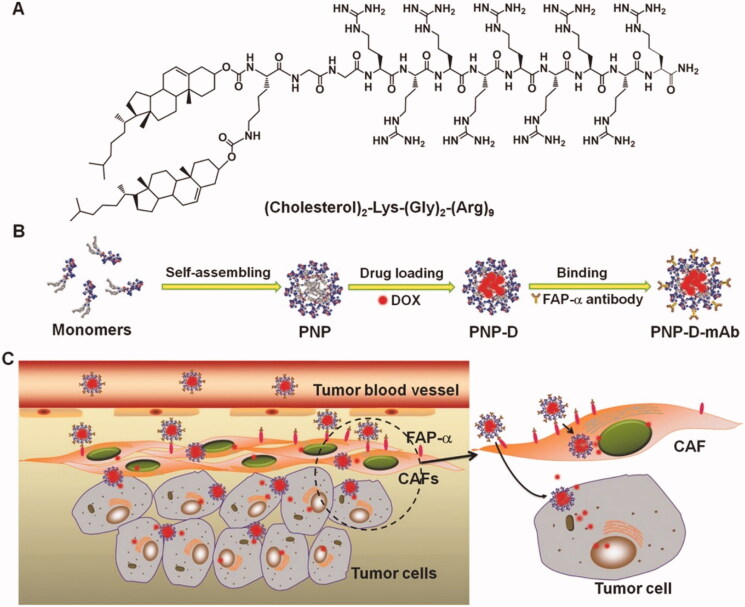
The design and the possible mechanism of PNP-D-mAb. (A) The structure of the cholesterol-modified CPP. (B) Schematic illustration of the nanoparticle formation process including peptide assembling, drug loading, and mAb modification. (C) The proposed mechanism of PNP-D-mAb in CAFs targeting and drug penetration. Figure reproduced from reference Ji et al. (2015).

Drug delivery is an advantageous and effective strategy for chemotherapy because it not only diminishes the drugs-related toxicity but also improves their target ability via passive or active transport. Inorganic nanomaterials, for example, core–shell structured materials or metal-organic framework materials have been used as drug carriers (Min et al., 2019). However, these nanomaterials suffer from several drawbacks, including toxicity and insufficient drug loading (Su et al., 2019). Therefore, it is a worthwhile strategy to discover new materials and optimize drug loading. Peptides have high biocompatibility and, more importantly, their amphiphilicity enables them to load hydrophobic therapeutic molecules during the self-assembling process, resulting in an increased drug loading rate. Peptides that self-assemble can form a variety of nanostructures, including rods, vesicles, and micelles, which facilitate cell uptake (Zhang et al., 2019a). As drug carriers, SAPs not only have a high rate of drug loading but also respond to the TME to make sure the drug is released properly. Nanoparticles based on the peptides can be employed for the effective loading of drugs such as doxorubicin (Ji et al., 2015). Based on the ability of peptides to be altered, monoclonal antibodies (mAbs) that could target human fibroblast activation protein-α were added to the surface of SAPs (PNP-D-mAb). Using the stimulus-responsive PNP-D-mAb in the TME (Figure 2), DOX was released at a rate of up to 80% for 48 h, while only 30% was released for 12 h, showing that DOX could be released effectively and controlled to release. NIR tumor imaging and tumor growth curves demonstrated that PNP-DmAb had a high degree of targeting ability and remarkable therapeutic efficacy. Additionally, peptides can self-assemble into fibrils or hydrogels, making them ideal for sustained drug delivery (Ren et al., 2019). Thus, SAPs as drug carriers have a high loading rate and high efficacy of drug release. Additionally, the novel strategy of recognition-reaction-aggregation is used in conjunction with addressable SAPs for increasing the sensitivity of chemotherapeutic agents and impairing cell membrane permeability (Li et al., 2019a). Importantly, SAPs as drug carriers improve targeting and decrease toxicity in comparison to free drugs, which is advantageous for future research and clinical application. Abraxane was the first medicine to use protein nanoparticles to load an anticancer drug, and it received FDA approval in 2005 (Cui et al., 2010). These efforts have been widely recognized, and in the future, an increasing number of drugs based on SAPs will enter clinical trials.

## 6. Recent developments in peptides-based nano-cargos

Significant advances have been achieved in anti-cancer drug delivery by utilizing a variety of techniques and nanocarrier systems to improve anti-tumor agent delivery to target sites. Selectivity for cancer can be achieved using a variety of novel DDSs that are either stimulus-sensitive or ligand-attached (Raza et al., 2019). Several promising drug delivery vehicles, such as nanoconjugates (NCs), nanoparticles, and vesicles have been developed (Lee et al., 2018; Yin et al., 2018; Du et al., 2020). Similarly, nano-targeted DDSs and intracellular smart NPs that respond to stimuli have been developed to deliver a combination of drugs in accordance with the TME (Kim et al., 2018; Li et al., 2018a; Xiong et al., 2019). Among them, pH and temperature are the most frequently used triggers among these stimuli. As a result, they are capable of specifically targeting, recognizing, and eliminating cancer cells, overcoming the limitation of low tumor selectivity. Similarly, several strategies have been developed to enhance the antitumor effect of SAPs. For example, SAPs have been reported as a TME sensitive drug delivery carrier (Moyer et al., 2014; Cao et al., 2019) or capable of active targeting (Xu et al., 2016; Du et al., 2018). To reactivate the suppressed immune response, immune cell epitopes and/or immune checkpoint inhibitors were conjugated with SAPs (Black et al., 2012; Li et al., 2021). Cytotoxic peptides conjugate SAPs (Standley et al., 2010; Toft et al., 2012), and self-assemblies of peptides induced by enzyme or pH in targeted organelle are lethal to cancer cells (Feng et al., 2018; Tan et al., 2021). To enhance the potential of photodynamic therapy (PDT), SAPs were conjugated with targeting peptide epitopes and photosensitizers (PSs) (Li et al., 2018c; Tian et al., 2021).

### 6.1. Targeted chemotherapeutic drug delivery

Chan investigated the potential of NPs to tumors in 2016, reporting a low accumulation (0.7%; median) of administered NPs in solid tumors (Wilhelm et al., 2016). After that they discovered that the accumulation efficiency of NPs delivered passively, via the enhanced effects toward the permeability and retention (EPR), is limited and tumor-specific, and that the active process of NPs to be transported into the tumor via endothelial cells is critical (Sindhwani et al., 2020). Various disadvantages are associated with peptide SA, such as specific targeting (Xu et al., 2016), released under the influence of stimuli (Cheng et al., 2018), increased penetration to cells (Moyer et al., 2014), and the capability of coordinating various therapies in a single-vehicle (Cheng et al., 2018). Therefore, SAPs have the potential to be used in the delivery of anti-cancer drugs (Dasgupta & Das, 2019). This section summarizes the chemotherapeutic loaded SAP-based nanostructures and their application in active targeting and environment-responsive drug delivery for cancer therapy.

**Figure 3.**
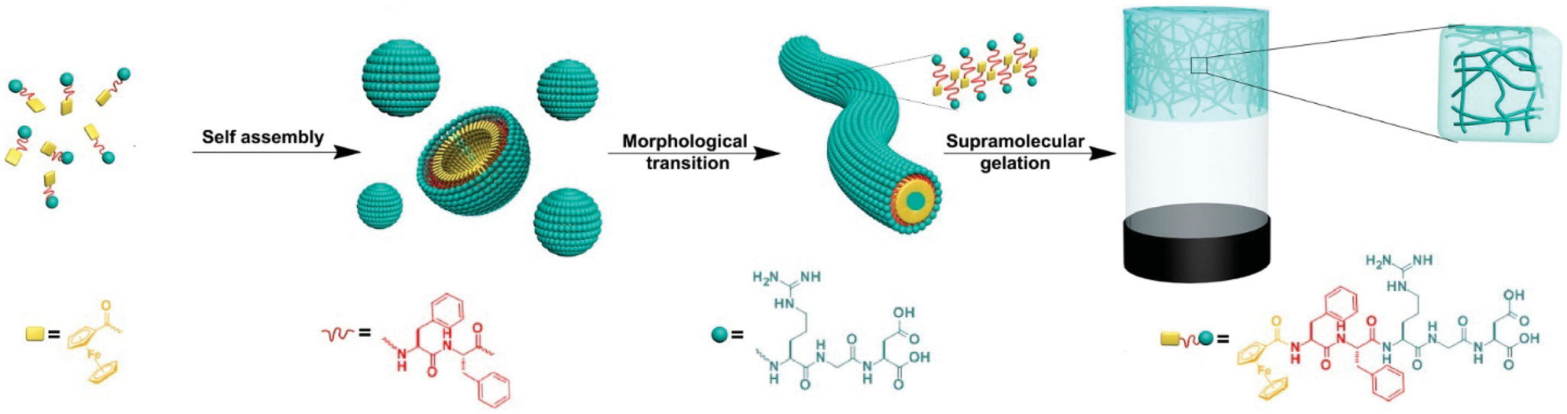
Fc-FFRGD self-assembling process for the production of supramolecular nanofibers and hydrogels. Reproduced with permission from ACS 2016 (Li et al., 2021).

Weak base containing peptides can provide a pH-responsive feature and are used in the design of assemblies capable to response the pH change. Cryns and Stupp et al. synthesized two PAs from a six His-based peptide sequence: C16H31O-H6-OEG (PA 1) and OEG-H6K-OC12H25 (PA 2). Both PAs self-assemble into nanofibers and spherical micelles, respectively (Moyer et al., 2014). At pH 7.5, the self-assemblies formed and disassembled at pH 6.0 enabling the control release of drugs, i.e. CPT. The pharmacokinetics and tissue distribution of PA 1 and PA 2 were significantly diverse. In comparison to PA 2-based nanoparticles, nanofibers based on PA 1 demonstrated a significantly higher level of blood circulation and a significantly greater amount of tissue accumulation. When it comes to *in vivo* assembly, peptide sequence determines everything from the morphology to the serum stability and tissue distribution. It also controls pH-triggered drug release. For anti-cancer drugs, PAs can also have the potential to respond to enzymes. Cao et al. developed Nap-FFGPLGLARKRK, a peptide derived from FF, for the delivery of anticancer drugs (Cao et al., 2019). The molecule consisted of the self-assembling motif (FF), the enzyme-sensitive peptide -GPLGLA-, and the positively charged -RKRK- segment that enhances the cell penetration. The combination of peptide and DOX yields two distinct types of nanofibrils with varying diameters. DOX could be released in tumors as a result of MMP7 overexpression in cancer cells. *In vivo* experiments revealed that the peptide/DOX composites effectively suppressed the growth of the tumor with reduced tumor metastasis sites in the liver (Figure 3).

Targeting peptides have a high affinity for tumor biomarkers (Xu et al., 2016). To construct nanovesicles, various potential building blocks have been used to specifically target the cancer cells, which include RGD peptides capable to interact with integrins, and the ATWLPPR peptide, which interacts with neuropilin-1 (Qin et al., 2017). Xu et al. designed an FF-derived peptide modified with RGD (Fc-FFRGD) (Xu et al., 2016). In an aqueous medium, the SA of Fc-FFRGD gives rise to metastable spherical particles, which were then transferred over two hours to an entangled nanofibers-based hydrogel. The RGD peptides present on the assembly surfaces induced DOX uptake into HeLa cells, revealing the benefit of incorporating targeting peptides into self-assemblies of the peptide. GE11, an EGFR-targeting peptide, was applied to PA-based self-assemblies by Yang et al. to co-deliver olaparib and gemcitabine for the treatment of pancreatic cancer (PCa) (Du et al., 2018). PCa cells have elevated levels of EGFR on their surface. Gemcitabine, first-line PCa chemotherapy, and olaparib, a poly-ADP-ribose polymerase inhibitor, were encapsulated in C18-(Glu)2-Gly-GE11 self-assembled nanoparticles (GENP). According to the findings, the targeting delivery of two chemotherapy drugs utilizing peptide self-assemblies is superior in treating PCa.

### 6.2. Cytotoxic peptide self-assemblies

**Figure 4.**
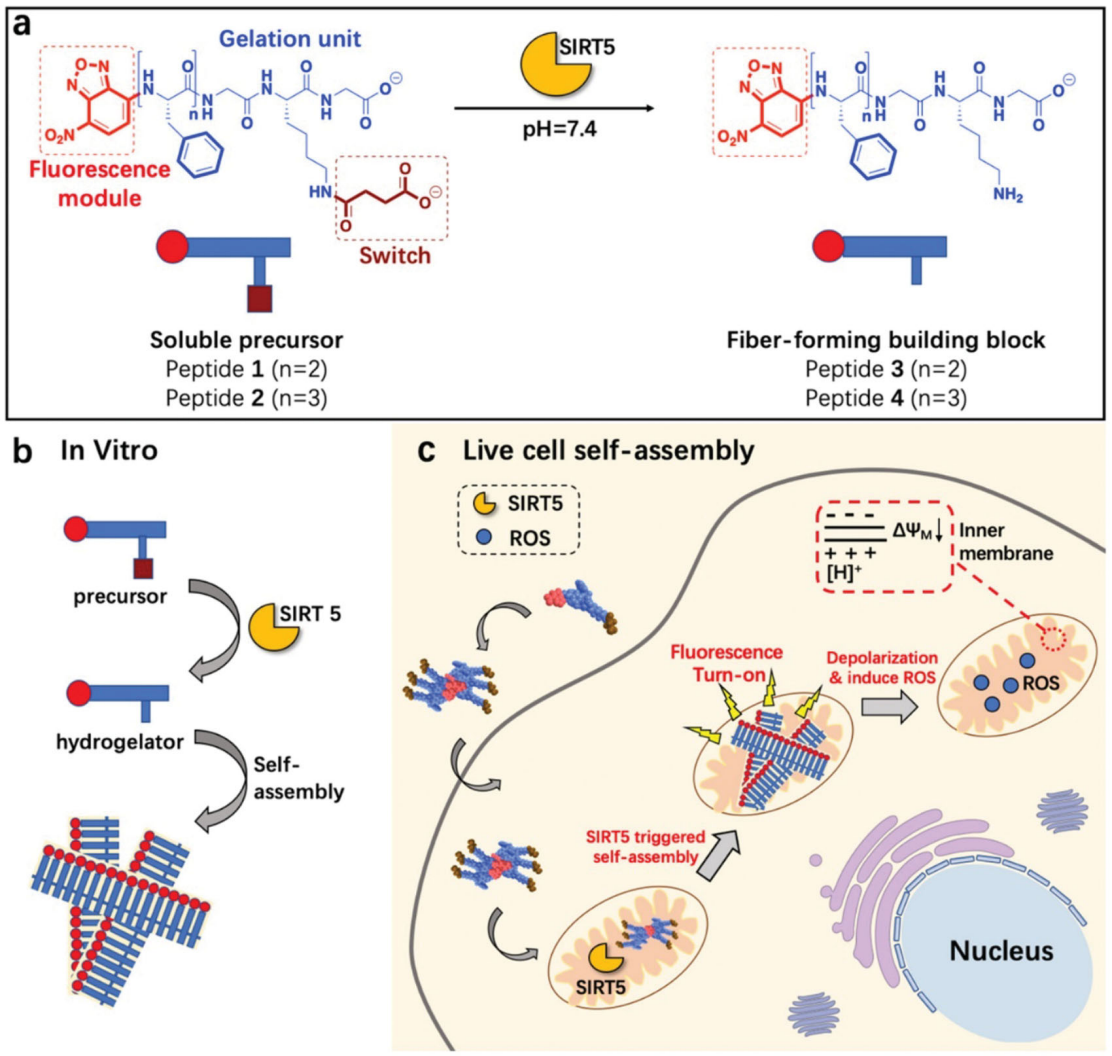
SIRT5-catalyzed mitochondria-restricted SA of peptide nanofibers. (a) Molecular structures of fiber-forming building blocks and precursors. SIRT5 can desuccinylate peptide precursors 1 and 2 to form the fiber-forming building blocks 3 and 4, respectively. (b) A schematic illustration of the SIRT5-induced SA mechanism *in vitro*. (c) Schematic illustration of mitochondrial intracellular fiber production via selective interaction of internalized peptide precursors with the SIRT5 enzyme. Reproduced with permission from ACS 2020 (Feng et al., 2017).

Cytotoxic peptides used as anticancer agents have several drawbacks, including poor cell permeation, immunogenicity, and proteolytic degradation (Chauhan et al., 2005). Cytotoxic peptides incorporation into SAPs is a promising strategy for resolving these issues. In research findings, it has been shown to increase therapeutic accumulation in tumors and thus boost anticancer efficacy without using any drugs. In 2010, a cationic α-helical (KLAKLAK)2 peptide was incorporated into a PA by Stupp et al. that undergo SA to form cylindrical nanofibers (Standley et al., 2010). α-helical conformation of KLAK domain as stabilized b the SA domain. The α-helical KLAK PA showed more effective tumor cell penetration than the scrambled KLAK PA, suggesting that KLAK PA nanostructures induced cell death via cell membrane disruption. Another approach for optimizing the performance of cytotoxic peptides includes the modification of the nanostructured surface with PEG, which inhibits the proteolysis, prolongs circulation time and reduces immune response (Joralemon et al., 2010). Based on the preceding work, Stupp and Cryns et al. developed a system that was based on co-assembly of pegylated PA (PEG PA) and the cytotoxic KLAK PA (Toft et al., 2012). PEG PA significantly reduced the degradation rate of KLAK PA, as demonstrated by the enzymatic degradation experiments, and this confirmed their co-assembly in the same nanostructure. Over four weeks, PEG PA exerted no negative effect on anticancer efficacy and was able to reduce breast cancer growth in a mouse model (Figure 4).

Xu's group invented enzyme-instructed self-assembly (EISA) enabling the *in situ* SA of SAPs under the influence of cancer-related enzymes, and the resulting nanostructures cause critical damage to cancer cells (Gao et al., 2010; Feng et al., 2018). Due to their high aggregation potential and ease of synthesis, FF derivatives, aromatic short peptides, have found widespread application (Feng et al., 2017). By conjugating the nanostructures with organelle-targeting functionalities, we can control the subcellular location of the nanostructures to develop cancer therapeutics. Additionally, pH-induced peptide SA in the cytoplasm has been considered a promising approach to treat cancer. Maruyama et al. developed a novel polymer, C16-VVAEEE, that self-assembles into nanofibers under the influence of a slight change in pH (Yamamoto et al., 2021). The pH-responsive SA is associated with acidic amino acid, whereas the alkyl chain is associated with the membrane or an organelle interaction that may cause the intracellular localization of Pas. At a relatively acidic pH (pH = 6.8), it formed a nanofiber-based hydrogel, but not at pH = 7. Hence, the lower intracellular pH within cells may be used to initiate *in situ* SA of C16-VVAEEE. The nanofibers generated in such conditions are associated with specific organelles interaction (for instance, the ER) and ultimately killed cells, as demonstrated by fluorescence imaging and *in vivo* results. Sun et al. described the *in situ* SA of a peptide-induced by an enzyme that significantly enhanced the anticancer activity of small drug molecules. It is composed of a Phe-rich fragment and a Ksucc (succinylated lysine) switch module denoted by the acronym Fmoc-FFFGKsuccG. (peptide 5). By using mitochondrial enzyme catalysis, supramolecular nanofibers have been developed via peptide precursors *in vitro* and living cells. Dichloroacetate (DCA), cisplatin (Cisplatin), and paclitaxel (Taxol), three commonly used chemotherapy drugs, kill cancer cells in different ways. Surprisingly, when peptide 5 and the three drugs worked together, they had a synergistic effect against HeLa cells. This research shows how to make new supramolecular nanosystems for SIRT5 imaging and anticancer therapy targeting mitochondria (Yang et al., 2020a).

### 6.3. Photodynamic therapy

**Figure 5.**
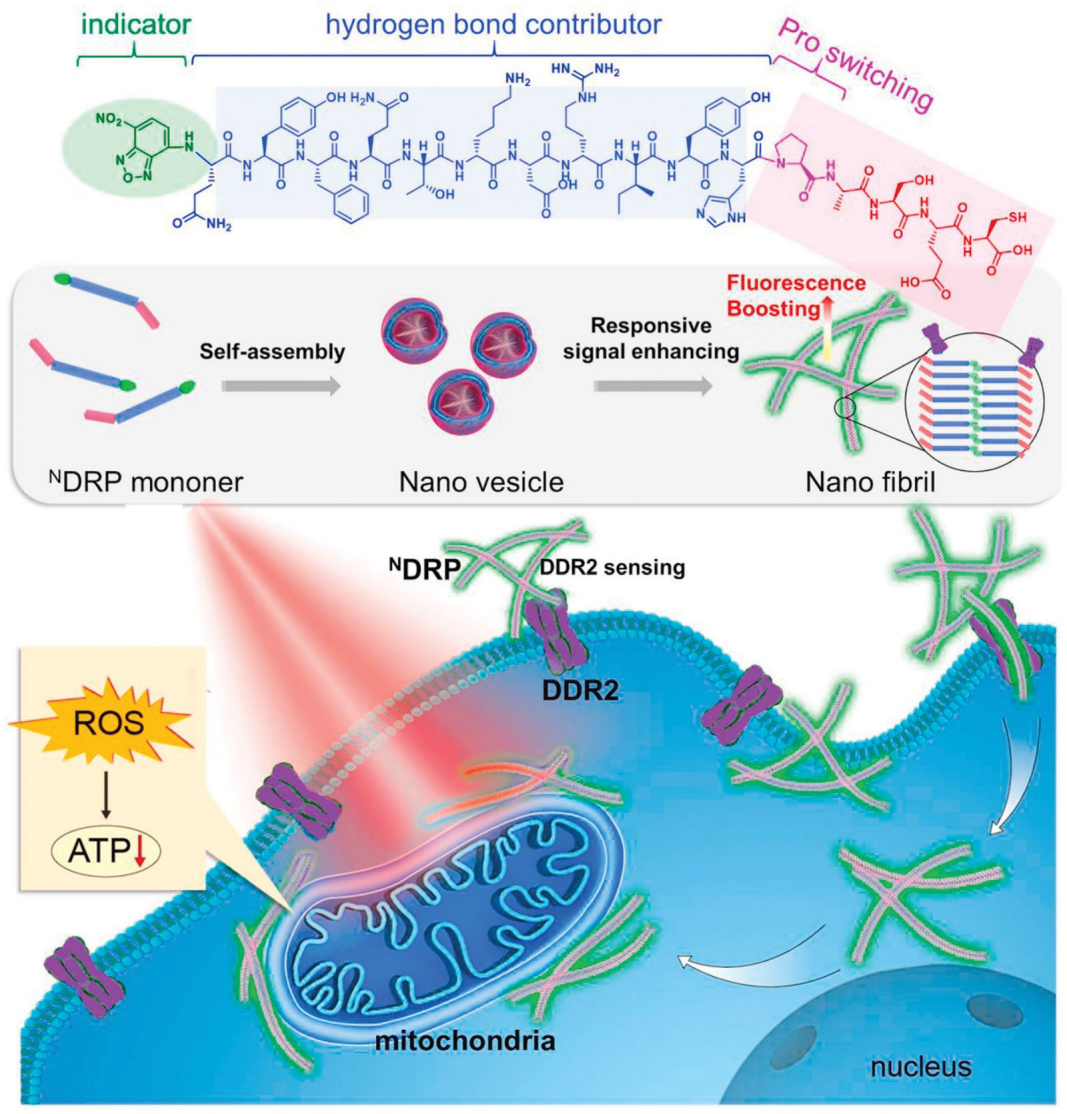
The nanosensor is depicted schematically in this diagram. ^N^DRP can be recognized and activated by DDR2, and then convert into a nanofibril and emit an increased fluorescence signal. Additionally, ^N^DRP may reach and destroy mitochondria. Figure reproduced with permission from reference (Wilhelm et al., 2016).

PDT employs singlet oxygen (1O2) produced by PSs in the presence of light to destroy tumors, has several practical drawbacks, including limited aqueous solubility, toxicity, and non-selectivity toward tumors (Liang et al., 2020; Wang et al., 2021a). To increase aqueous solubility, targeted delivery, and biocompatibility, PSs can be conjugated with SAPs. It is also possible to boost the effectiveness of PDT treatment by using specific peptide epitopes like iRGD or ERGD (Jiang et al., 2018; Zhu et al., 2019). Peptides and PSs can be co-assembled directly via hydrophobic interactions and π–π stacking. Bai reported the co-assembly of an aromatic short peptide Fmoc-Leu3-OMe with porphyrin derivative meso-tetra(p-hydroxyphenyl)porphine (m-THPP), resulting in the formation of photodynamic nanoparticles, nanoPSs (Li et al., 2018b). Porphyrin aggregation was prevented by the surrounding peptides. The data obtained by the cytotoxicity assay suggested that with light irradiation, the nano PSs displayed more obvious toxicity than when not irradiated. The tumors of nano-PSs-treated mice were inhibited and eradicated within two weeks of treatment, according to *in vivo* experiments (Figure 5).

Wang et al. also developed NDRP, a morphologically transformable peptide SA-fluorophore platform capable of targeting and damaging mitochondria for tumor theranostics (Tian et al., 2021). ^N^DRP is composed of a fluorophore NBD with hydrophobic responsive behavior, a novel peptide sequence DRP, and a module for morphological modification. ^N^DRP can be induced by the mesenchymal tumor marker DDR2 and subsequently converted *in vitro* from nanovesicles to nanofibers. The cationic hydrophilic part of nanofibril and ^N^DRP lipophilicity has a role in mitochondrial targeting. There was a good overlap between the ^N^DRP fluorescence signals and the MitoTracker (a mitochondrial marker), indicating that the mitochondrial-targeting effect had been observed. ^N^DRP-based nanostructures encapsulated the PS, Ce6, inhibiting the tumor growth in the MDA-MB-231 xenograft tumor-bearing mice. This work produced a multi-stage nanosensor device for cancer diagnosis and PDT.

### 6.4. Immunotherapy

**Figure 6.**
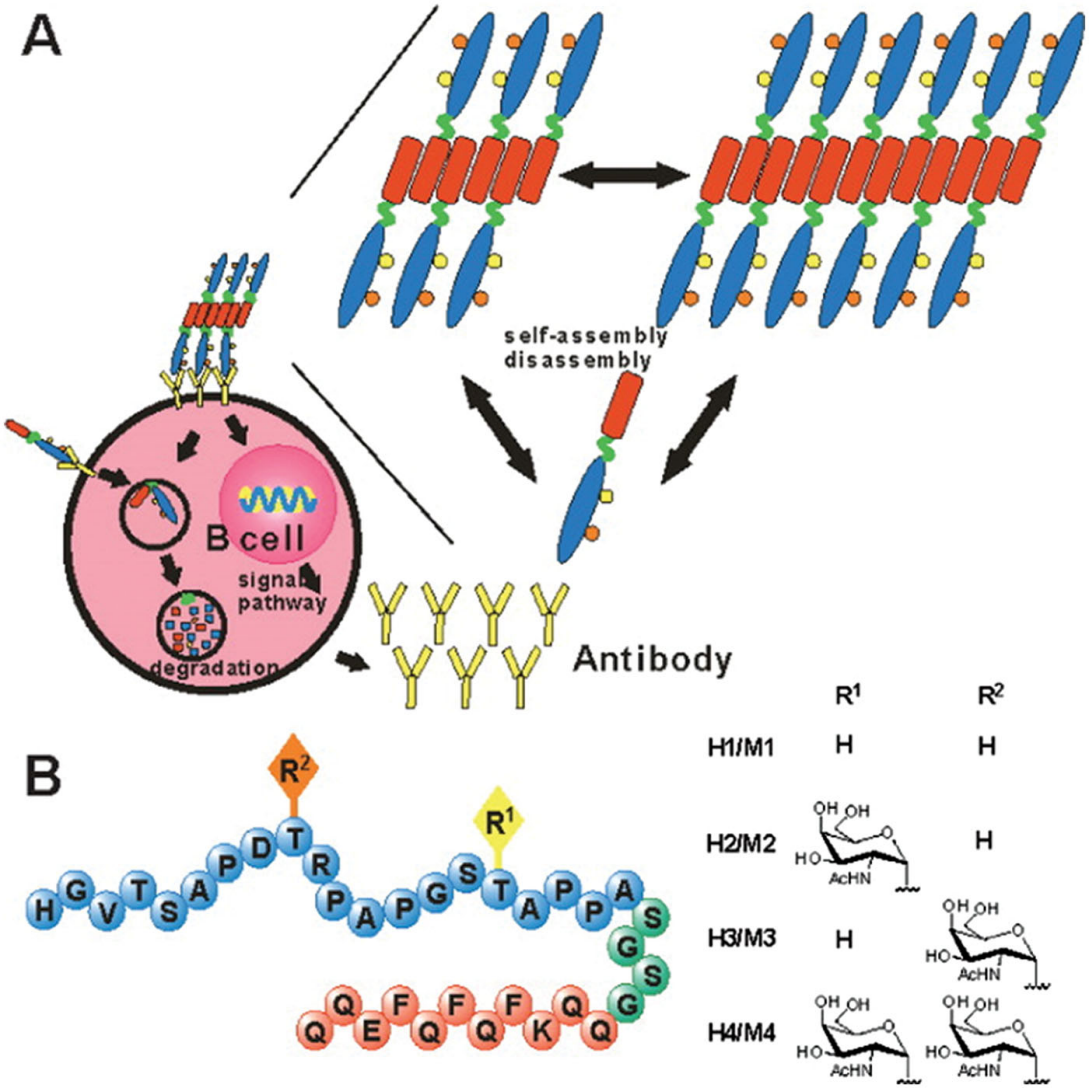
H1, H2, H3, and H4 self-adjuvant vaccine design. (A) Vaccine candidates can self-assemble into fibers and activate B cells. (B) The vaccination candidates H1, H2, H3, and H4 comprise the MUC1 VNTR's 20-mer B-cell epitopes M1, M2, M3, and M4 (peptide sequence in blue). Each vaccination contains a unique MUC1 glycosylation pattern (R, yellow, and orange, with R moieties specified on the right), a SA Q11 domain (red), and a flexible spacer (green). Reproduced with permission from ACS 2012 (Darvin et al., 2018).

Cancer cells frequently suppress the immune system in the tumor environment, preventing it from performing effective antitumor responses (Froimchuk et al., 2020). To address some of the shortcomings of conventional immunotherapy, SAPs have emerged as a promising technology that offers numerous advantages including efficient loading of cargo, multivalent antigen presence, and enhanced cellular uptake (Cai et al., 2020; Froimchuk et al., 2020). Tirrell et al. used PAs to synthesize cylindrical micelles with a cytotoxic T-cell epitope on the periphery (Black et al., 2012). Multiple antigen exposure to the cylindrical diC16-OVA micelles induced an immunological response even without the inclusion of an adjuvant. Certain peptides capable of forming short fiber, such as Q11 and RADA-16, have the potential to act as self-adjuvanting vaccine platforms. Li et al. developed several vaccine candidates using glycosylated B-cell epitopes in conjunction with the Q11 domain (Huang et al., 2012). The surfaces of long fibrils formed via peptide-containing B-cell epitopes, aggregating into long chains. Immunological studies revealed that the B cell epitope-Q11 SA generated an immune response, and the resulting antibody detected human MUC1-expressing tumor cells. MCF-7 cells were killed by the complement-dependent cytotoxicity mediated by one of the self-adjuvanting peptides (H4) (Figure 6).

One of the most significant advances in cancer immunotherapy has been the inhibition of immune checkpoints, such as PD-1 and PD-L1 inhibitors that have shown promising results against cancer (Darvin et al., 2018). However, because of their relatively larger molecular weights, clinical immune checkpoint blockers (ICBs) tend to have limited penetration. Another issue is developed resistance as a result of the frequent use of ICBs (Gide et al., 2018). Li et al. recently developed a tri-functional SAP to address these issues (Rehman et al., 2021). Here, three distinct functional domains were combined into a single short peptide sequence indoleamine 2,3-dioxygenase (IDO), an enzyme that is highly expressed and involved in the inhibition of effector T lymphocytes. The IND domain functions as an IDO inhibitor, reversing IDO-mediated immunosuppression; the G^D^F^D^F^D^Y domain acts as a powerful adjuvant carrying immunostimulatory characteristics, and the short peptide DPPA-1 domain serves as an antagonist to the PD-1/PD-L1 pathway. When compared to the control groups, the self-assembled―trident‖, IND-GDFDFDY^D^-PPA-1, proved to be the most effective in reducing tumor development and inhibiting tumor metastasis, as compared to control groups. All the three domains worked together to activate and infiltrate cytotoxic CD8+ T lymphocytes (CTLs) in tumors, and release perforin which in turn induced tumor cell death.

## 7. Conclusions and prospects

Peptide-based DDSs that can be self-assembled are being used in cancer diagnostics and treatment. To achieve high selectivity and specificity, nanomedicines are being functionalized to target tumors and minimize systemic toxicity. TME has a significant impact on the efficacy of cancer therapy. Cancer nanomedicine's behavior is governed by the TME. Cancer nanomedicine research, particularly self-assembled peptide hydrogels, has resulted in numerous improvements in cancer diagnosis and treatment. The focus of nanotherapeutics is expected to expand in the coming years as more medical concerns such as organ toxicity and relapse become apparent. Nanocarriers will provide additional benefits to cancer therapies because of their flexible structure and design of nanomaterials. Due to their chain lengths and unique sequences, self-assembled peptides form organized network structures such as hydrogels. They possess high sensitivity toward TME. Despite the numerous advantages, some challenges remain that should be addressed in the future for SAPs against malignant tumors.

If we consider protein to be the highest level of nanomaterials based SAPs, we can still develop various interesting substances to mimic the protein, such as (i) uniform chiral SAs; (ii) dynamic and reversible assemblies *in situ*; (iii) precise control over the order in which bioactive motifs are arranged; and (iv) dynamic modulation of bioactivities depending on biomarkers. To achieve this, we may need to understand the two gaps, from molecules to self-organization and medical applications, specifically the formation of self-assemblies and their interactions. The thermodynamics and kinetics of SA of SAPs explain the sequence and condition effects, hierarchical assembly, and energy landscape. The association between sequence and morphological characteristics of SA for short peptides (*n* = 2 and 3) has become increasingly obvious with the advancement of computer simulation. However, for larger peptides, when the number of possible sequences increases exponentially (n20), it becomes extremely difficult to screen all conceivable sequences. In such conditions, systematic investigations of SAPs composed of a limited number of AA types provide a small insight into the sequence effect. Recently, a surge was observed in interest in chiral SAs, such as helical structures. SAPs are a great option for chiral assemblies due to their both enantiomers (L and D), which have already demonstrated the usage in templates, chiroptics, separation, and chiral sensing. The general principle behind the formation of chiral assemblies from SAPs remains unknown. It is difficult to obtain unified chiral assemblies, and their use in biomedicine is still rare. The cell-to-SA interactions are another area where there is a need for improvement. This dynamic attribute will have an effect on the stability of SA within the body and, consequently, on performance. At the molecular level, the dynamic interaction of SAP-based SAs with the cell membrane remains a mystery.

SAP-based hydrogels exhibit low mechanical strength for application purposes. Covalent crosslinking techniques, such as enzymatic crosslinking or oxidation, are effective methods for improving the mechanical properties of composite materials. Non-covalent crosslinking techniques, such as incorporation with inorganic nanoclay, polymers, and nucleic acids, offer an alternate method for increasing mechanical characteristics. Co-assembly of numerous components is also necessary to combine multiple functionalities for a synergistic impact worth exploiting for improved therapeutic or diagnostic performance. Developing suitable nanomaterials based on SAPs for certain types of tumors using controlled techniques to introduce new insights toward tumor treatment. More crucially, altering the peptide chains is usually carried out with other materials to protect the peptides from enzyme degradation in physiological conditions and to avoid peptide immune response *in vivo*. In conclusion, an increasing number of functional peptides are being used in tumor theranostics. In the near future, we expect that SAPs, particularly functional peptides, will have significant practical value in biological and medicinal sites (Kuang et al., 2018).
